# Glutathione for skin lightening via intravenous, oral, and topical routes: a critical narrative review of current evidence, safety, and the evidence-practice gap

**DOI:** 10.25122/jml-2026-0054

**Published:** 2026-07

**Authors:** Khawlah Aloraini, Abdulrahman Alangari, Jamal Arif, Fahad Alkhodairy

**Affiliations:** 1 Department of Medicine, evercare, Riyadh, Saudi Arabia; 2 Department of Research & Development, evercare, Riyadh, Saudi Arabia; 3 Department of Gastroenterology and Hepatology, Prince Sultan Military Medical City, Riyadh, Saudi Arabia; 4 College of Medicine, Shaqra University, Shaqra, Saudi Arabia

**Keywords:** glutathione, intravenous, skin lightening, vitamin C, melanin, topical GSH, oral GSH, skin depigmentation, GSH, reduced glutathione, GSSG, oxidized glutathione, IV, intravenous, MITF, microphthalmiaassociated transcription factor, RCT, randomized controlled trial, ROS, reactive oxygen species

## Abstract

Intravenous glutathione (IV GSH) has emerged as a prominent cosmetic skin-lightening treatment despite continuing uncertainty about its efficacy and safety. This narrative review critically appraises the available evidence on IV GSH, with special emphasis on its proposed mechanisms of action, clinical efficacy, safety profile, and knowledge gaps. We performed a structured literature search using PubMed/MEDLINE, Scopus, Embase, and Google Scholar from inception through March 2026. We screened and included mechanistic, preclinical, and clinical trials assessing GSH for skin lightening in the final narrative synthesis. The biological hypothesis for GSH is that it can block the tyrosinase pathway, influence oxidative stress regulation, and direct melanogenesis toward pheomelanin synthesis. However, translating these mechanisms into clinically meaningful outcomes remains uncertain. Available literature provides limited evidence of modest, varied skin pigmentation improvements and is generally limited by small sample sizes, methodological differences, the absence of standardized outcomes, and limited follow-up. In addition, the regular co-administration of vitamin C has made it difficult to know the independent effect of IV GSH in such individuals. New safety concerns, such as hepatotoxicity, hypersensitivity reactivity, and potential renal and endocrine effects, may further complicate the risk–benefit relationship during IV GSH. Current evidence is too limited to justify routine use for cosmetic skin lightening, highlighting the necessity for sufficiently powered RCTs and prolonged safety studies.

## INTRODUCTION

Skin pigmentation is one of the most visible human phenotypic characteristics and is primarily determined by the quantity, type, and distribution of melanin within the epidermis. Melanin is synthesized by melanocytes and subsequently transferred to surrounding keratinocytes through melanosomes [1–3]. Two principal forms of melanin exist: eumelanin, which produces brown to black pigmentation, and pheomelanin, which imparts yellow to reddish hues. The relative proportions of these pigments, together with total melanin content, largely determine constitutive skin color [3,4].

The global demand for skin-lightening interventions has grown markedly over the past 20 years. This trend has emerged due to complex social and cultural factors that contribute to the pursuit of beauty (the perceived beauty of lighter skin tones) as well as the social status (the upward mobility in opportunities) linked to lighter skin tones [5–7]. As a result, significant commercial demand has developed for depigmenting agents, from commercial topical therapies (e.g., hydroquinone, kojic acid, azelaic acid, and arbutin [3,8]) to systemic therapies (e.g., oral or intravenous [IV] glutathione [GSH] [9–11]).

GSH is an endogenous tripeptide that consists of glutamate, cysteine, and glycine and is one of the main intracellular antioxidants in human cells. Beyond its predominant role in protecting cellular redox balance, GSH also supports detoxification mechanisms and several enzymatic reactions necessary for cellular homeostasis [12,13]. GSH was considered a dark skin-lightening agent after its role in melanogenesis was demonstrated through inhibition of tyrosinase activity, scavenging of reactive oxygen species (ROS), and diversion of eumelanin-based melanin synthesis to pheomelanin synthesis [3,14,15].

Despite this biologically plausible rationale, the rapid adoption of GSH, especially IV GSH, in esthetic practice has been implemented ahead of a broad therapeutic landscape, with substantial clinical research to support compliance and regulatory intervention [9–11]. IV GSH has been widely encouraged in cosmetic clinics because it theoretically provides the most systemic bioavailability with no involvement in gastrointestinal metabolism [16–20]. Nonetheless, the evidence to support these regulations has drawn more concern about its efficacy, safety, and long-term effects and has caused a series of authoritative regulatory agencies, such as the Philippine Food and Drug Administration [21] and the Saudi Food and Drug Authority [22], to sound a warning about its use in cosmetic skin lightening [10,11,18,19,23].

Importantly, clinical evidence is limited and varied. Most of these studies report small sample sizes, short treatment durations, variable dosing protocols, different outcome measures, and a lack of long-term safety evaluation [11,24]. In addition, multiple clinical trials have combined GSH with vitamin C, making it challenging to isolate the contribution of GSH in clinical settings [11,19].

While earlier narrative and systematic reviews reviewed GSH supplementation by route of administration, our review is more critical and focused on clinical assessment. IV GSH received particular emphasis because it represents the most evidence–practice gap formulation: widespread use of this cosmetic treatment despite limited high-quality evidence and a lack of ongoing safety assessment. This review includes oral and topical GSH reports to provide comparative context on bioavailability, efficacy, and safety.

By integrating mechanistic insights, clinical evidence, comparative route-specific data, and safety considerations, this review aims to provide a balanced and clinically relevant appraisal of GSH-based skin-lightening interventions. Specifically, it addresses the pertinent clinical question of whether plasma concentrations increased by IV administration, leading to superior clinical outcomes, are associated with improved symptoms compared with oral and topical formulations, and highlights the knowledge gaps that must be addressed before empirical evidence-based recommendations can be developed.

## SEARCH STRATEGY AND STUDY SELECTION

This review was conducted as a structured narrative review to explore and critically assess the current evidence on the use of GSH for skin lightening, predominantly evaluating IV GSH, as well as evidence for oral, topical, and other formulations providing comparative information on efficacy, bioavailability, and related safety. The methodology was intended to provide a transparent and comprehensive assessment of the literature and acknowledges the considerable heterogeneity of the evidence, as well as the limited number of high-quality clinical studies.

We performed a comprehensive electronic literature search using PubMed/MEDLINE, Scopus, Embase, and Google Scholar from database inception through March 2026. The search strategy combined Medical Subject Headings (MeSH), Emtree terms, and free-text keywords related to GSH and pigmentation biology. The principal search terms included “glutathione,” “GSH,” “intravenous glutathione,” “IV glutathione,” “oral glutathione,” “topical glutathione,” “sublingual glutathione,” “skin lightening,” “skin whitening,” “depigmentation,” “melanogenesis,” “tyrosinase,” “melanin,” “pheomelanin,” “eumelanin,” “vitamin C,” “ascorbic acid,” and “adverse effects.” Boolean operators (“AND” and “OR”) were used to optimize search sensitivity, and database-specific search strategies were adapted according to indexing requirements.

The electronic search yielded 431 records: 154 from PubMed/MEDLINE, 89 from Scopus, 76 from Embase, and 112 from Google Scholar. Following automated and manual removal of duplicate publications, 407 unique records remained for title and abstract screening. Two reviewers independently screened all retrieved records for relevance, and potentially eligible studies subsequently underwent full-text assessment.

Studies were considered eligible if they (i) evaluated IV GSH for cosmetic skin lightening or pigmentary disorders; (ii) investigated oral, topical, or other GSH formulations that could provide comparative clinical or pharmacological information relevant to IV administration; or (iii) provided mechanistic, pharmacokinetic, or safety data relevant to GSH-mediated effects on melanogenesis and pigmentation. Randomized controlled trials (RCTs), non-randomized interventional studies, observational studies, pilot studies, case series, mechanistic investigations, and relevant review articles were considered for inclusion if they provided meaningful evidence relevant to the objectives of this review.

Studies were excluded if they were non-English publications without accessible translations, conference abstracts lacking full-text availability, duplicate publications, editorials, letters, commentaries, or publications that did not report relevant pigmentation outcomes or provide mechanistic information related to melanogenesis and GSH biology.

**Figure 1:**
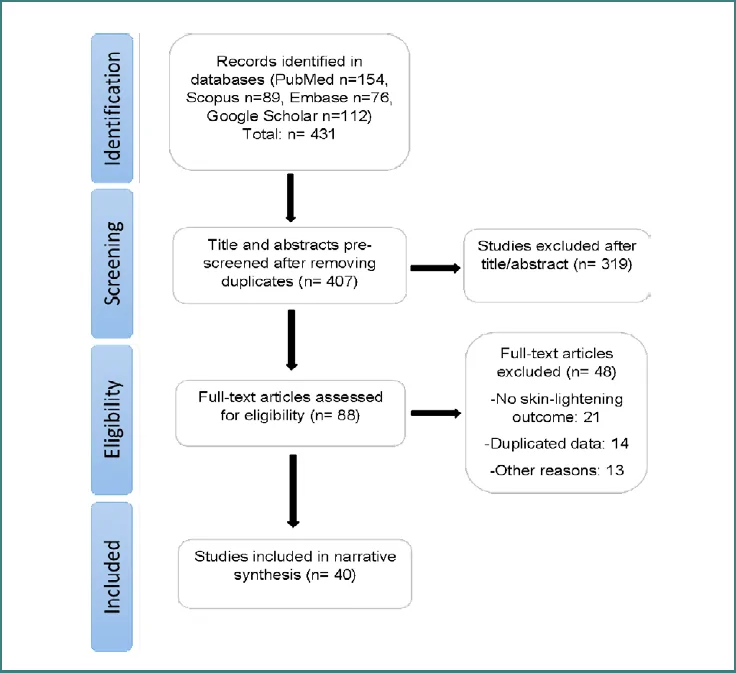
PRISMA-style flow diagram illustrating the study identification, screening, eligibility assessment, and inclusion process for this narrative review. Of 431 records identified across four databases, 40 studies were ultimately included in the narrative synthesis

Following title and abstract screening, 88 articles were retrieved for full-text evaluation. Of these, 48 studies were excluded because they lacked quantifiable pigmentation outcomes (*n* = 21), represented duplicate data from previously included cohorts (*n* = 14), or consisted of editorials, letters, and other non-peer-reviewed publications (*n* = 13). Forty studies met the eligibility criteria and were included in the final narrative synthesis (Figure 1). To ensure full literature coverage, we manually screened the reference lists of all included studies and pertinent review articles to identify additional eligible studies not captured through electronic database searching.

### Data extraction

Two reviewers independently extracted data using predefined, standardized data collection guidelines. When feasible, the following details were extracted: first author, year of publication, country of origin, study design, participant characteristics, GSH formulation and dosage, route of administration, treatment duration, concomitant interventions (especially vitamin C administration), methods used for pigmentation assessment, efficacy outcomes, adverse events, and major study limitations. We resolved discrepancies in data extraction through discussion and consensus.

### Assessment of study quality

Given the wide scope of this review and the heterogeneous study designs, including RCTs, observational studies, and mechanistic studies, a single formal risk-of-bias assessment tool was not uniformly applicable. However, we critically examined methodological quality during data interpretation, including but not limited to study design, sample size, use of control groups, duration of follow-up, objective assessment of pigmentation outcomes, completeness of adverse-event reporting, and potential sources of bias. Greater weight was assigned to findings derived from RCTs, whereas evidence from uncontrolled studies, observational investigations, and mechanistic studies was interpreted more cautiously.

### Data synthesis

Because of substantial heterogeneity in study populations, GSH formulations, dosing regimens, concomitant therapies, outcome measures, and follow-up durations, quantitative pooling of data and formal meta-analysis were considered inappropriate. Hence, we used a narrative synthesis approach. Based on the route of GSH administration, study design, efficacy outcomes, safety findings, and overall strength of evidence, we critically appraised and synthesized all evidence, with special emphasis on clinical applicability, methodological limitations, and areas requiring further investigation. While the current review targets IV GSH, evidence on oral and topical formulations was purposefully included to enable comparison of delivery processes across routes of administration and to provide context for current evidence on IV GSH.

## BIOCHEMICAL BASIS OF GSH IN MELANOGENESIS

Several interconnected biochemical mechanisms support the proposed depigmenting effects of GSH by influencing both melanin synthesis and cellular redox homeostasis. Melanogenesis begins with the conversion of L-tyrosine to L-3,4-dihydroxyphenylalanine (L-DOPA), followed by the oxidation of L-DOPA to dopaquinone, reactions that are catalyzed by tyrosinase, the rate-limiting enzyme in melanin synthesis [1,3,15]. Dopaquinone subsequently serves as the key branch-point intermediate from which either eumelanin or pheomelanin is generated, depending largely on the availability of sulfhydryl compounds such as cysteine and GSH [3,25,26].

**Figure 2:**
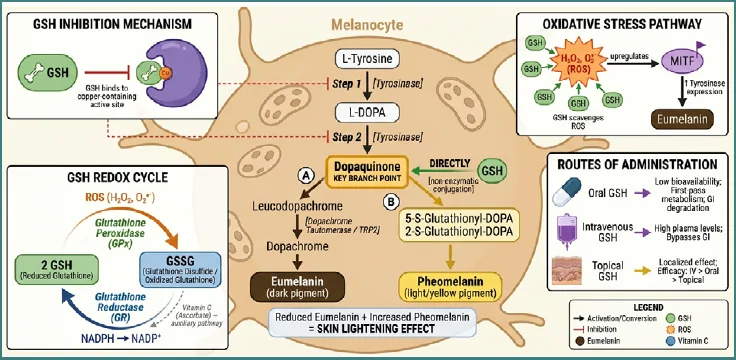
Schematic representation of the proposed mechanisms by which GSH exerts skin-lightening effects through melanogenesis modulation and oxidative stress pathways. GSH inhibits tyrosinase at its copper-containing active site (Step 1, L-tyrosine **→** L-DOPA; Step 2, L-DOPA **→** dopaquinone) and diverts dopaquinone, the key branch-point intermediate, toward pheomelanin via direct, non-enzymatic conjugation to 5-S-glutathionyl-DOPA and 2-S-glutathionyl-DOPA (Path B), while suppressing eumelanin synthesis through the leucodopachrome **→** dopachrome route (Path A, catalysed by TRP2). GSH also scavenges reactive oxygen species (ROS; H_2_O_2_, O_2_•**⁻**), reducing oxidative-stress-driven upregulation of MITF and tyrosinase. In the redox cycle, 2 GSH are oxidized to GSSG by GPx and regenerated by GR using NADPH (NADPH **→** NADP**^+^**), with ascorbate (vitamin C) as an auxiliary non-enzymatic route. Abbreviations: GPx, glutathione peroxidase; GR, glutathione reductase; GSH, reduced glutathione; GSSG, oxidized glutathione; MITF, microphthalmia-associated transcription factor; NADPH, nicotinamide adenine dinucleotide phosphate; ROS, reactive oxygen species; TRP2, tyrosinase-related protein 2.

GSH appears to influence melanogenesis through several complementary pathways (Figure 2). First, GSH can directly inhibit tyrosinase activity by interacting with the enzyme’s copper-containing catalytic site, thereby reducing the conversion of tyrosine and L-DOPA into downstream melanogenic intermediates [14,15]. Second, GSH reacts non-enzymatically with dopaquinone to form glutathionyl-DOPA conjugates, including 5-S-glutathionyl-DOPA and 2-S-glutathionyl-DOPA, which favor pheomelanin synthesis at the expense of eumelanin production [25,27]. This shift toward the formation of lighter pigment species is one of the key mechanisms proposed to explain some clinical findings of skin-lightening associated with its effect.

Besides its direct activity on melanogenic enzymes, GSH exerts important antioxidant actions that may indirectly influence pigmentation. Oxidative stress is central to melanocyte activation, and ROS, such as hydrogen peroxide and superoxide radicals, have been demonstrated to induce tyrosinase activity and melanogenesis [28]. GSH may neutralize oxidative stress-induced activation of melanin synthesis by scavenging these reactive species and maintaining intracellular redox balance. Moreover, intracellular GSH levels affect signaling pathways involved in melanocyte function, including the regulation of microphthalmia-associated transcription factor (MITF) and tyrosinase-related proteins, both of which are primary regulators of pigment production [28,29]. Accordingly, the anti-melanogenic potential of GSH should instead be understood in the context of cellular redox biology. It participates in the intricate network of antioxidant and signaling pathways that collectively influence melanocyte activity and pigment production [28,29]. The interplay between direct enzymatic inhibition, diversion of melanogenesis toward pheomelanin synthesis, and attenuation of oxidative stress provides a biologically plausible explanation for GSH’s observed depigmenting effects.

Experimental studies further support this mechanistic framework. Benathan [30] and Benathan *et al*. [31] demonstrated that intracellular thiol status, particularly the availability of cysteine and GSH, plays a crucial role in regulating melanogenic pathways and determining the balance between eumelanin and pheomelanin production. These studies suggested that higher intracellular GSH concentrations favor pheomelanogenesis and may suppress overall melanin synthesis in a concentration-dependent manner. Additional *in vitro* and experimental evidence indicates that exogenous GSH may modulate ultraviolet (UV)-induced melanocyte activation. Nagapan *et al*. [32] demonstrated that systemic GSH administration influences redox homeostasis in experimental models exposed to UVB radiation, supporting the view that GSH serves as a member of an overall antioxidant defense network rather than an isolated depigmenting substance.

GSH pharmacokinetics further influence its potential biological effects. Oral GSH undergoes substantial degradation within the gastrointestinal tract and is subjected to first-pass hepatic metabolism, resulting in variable and relatively limited systemic bioavailability [33–35]. Nevertheless, prolonged oral supplementation has been shown to increase systemic GSH stores, suggesting that at least a proportion of administered GSH or its metabolites becomes biologically available [36]. In contrast, IV administration bypasses gastrointestinal and hepatic metabolism and achieves substantially higher circulating GSH concentrations. Handog *et al*. [18] similarly noted that IV administration produced markedly higher plasma levels than oral or buccal formulations, theoretically increasing substrate availability for interaction with melanocytes.

Despite these mechanistic and pharmacokinetic considerations, biological plausibility should not be equated with proven clinical efficacy. The direct influence of elevated systemic GSH concentrations, particularly after IV administration, on melanocyte activity *in vivo* is uncertain. Melanogenesis is influenced by a diverse array of genetic, hormonal, inflammatory, and environmental determinants, and it remains unclear whether pharmacological elevations of circulating GSH via IV injection are sufficient to produce consistent, clinically meaningful depigmenting effects [17,23,37,38]. This uncertainty probably accounts, in part, for the discrepancy between the compelling mechanistic rationale and the modest and variable clinical outcomes reported in human studies.

### Clinical evidence and current state of evidence for GSH-based skin-lightening interventions

The existing literature on the benefit of IV GSH for skin lightening spans a wide range of studies, including preclinical animal studies and uncontrolled pilot studies, to a small number of RCTs. There needs to be adequate contextualizing of each study for its methodological rigor: animal studies can provide mechanistic information, but are not necessarily applicable to a translational perspective; pilot studies with no control groups are susceptible to regression to the mean and placebo effects; and limited RCTs also exist, despite presenting the highest degree of clinical evidence in this field because of small number of participants and short duration. Biologically plausible, these theoretical mechanistic claims should not be confused with experimental evidence of actual clinical outcomes. The prevalence of IV GSH in esthetic practice, outside regulatory approval and despite a lack of meaningful clinical evidence, illustrates the disconnect between clinical need and evidence-based medicine (Table 1).

**Table 1:** Clinical studies evaluating GSH for skin lightening

Study (Year)	Study Design	Population (n)	Route of Administration	Intervention	Duration / Follow-up	Outcome Measures	Key Findings	Major Limitations	Level of Evidence
Katipunan & Villafuerte (2011)	Pilot prospective study	Filipino adults (*n* = 21)	IV	GSH 600 mg weekly	10 weeks	Melanin index, physician assessment	Significant reduction in melanin index during treatment period	No control group, small sample size, short follow-up	Low
Zubair *et al*. (2016)	Randomized placebo-controlled trial	Pakistani adults seeking skin lightening (*n* = 32)	IV	GSH 1200 mg plus vitamin C twice weekly	6 weeks	Visual pigmentation assessment, patient satisfaction	Improvement reported in approximately 37.5% of treated participants	Small sample, concomitant vitamin C confounding, subjective outcomes	Moderate
Arjinpathana & Asawanonda (2012)	Randomized double-blind placebo-controlled trial	Healthy volunteers (*n* = 60)	Oral	GSH 500 mg/day	4 weeks	Melanin index (Mexameter), skin color assessment	Modest but statistically significant reduction in melanin indices, particularly at sun-exposed sites	Short treatment duration and uncertain long-term significance	Moderate
Handog *et al*. (2016)	Open-label, single-arm study	Filipino women (*n* = 30)	Oral (lozenge/buccal preparation)	Novel GSH formulation	8 weeks	Melanin index, skin assessment	Significant but modest reduction in pigmentation and acceptable tolerability	No placebo control, open-label design, limited follow-up	Low
Watanabe *et al*. (2014)	Double-blind placebo-controlled RCT	Healthy women (*n* = 30)	Topical	GSSG lotion	10 weeks	Skin brightness, wrinkles, moisture, melanin index	Improvement in skin brightness and overall skin condition	Small sample size; topical formulation only	Moderate
Wahab *et al*. (2021)	Double-blind randomized controlled trial	Adults with facial hyperpigmentation (*n* = 46)	Oral + Topical	Combined oral and topical GSH therapy	12 weeks	Melanin index, colorimetry, physician assessment	Combination therapy showed superior efficacy compared with monotherapy	No IV comparator arm; relatively short follow-up	Moderate
Duperray *et al*. (2022)	Randomized double-blind benchmark- and placebo-controlled trial	Adults with skin hyperpigmentation (*n* = 124)	Oral	L-cystine associated with GSH (250–500 mg/day equivalent formulation)	12 weeks	Individual Typology Angle (ITA°), skin brightness and pigmentation measurements	Significant improvement in skin pigmentation and brightness compared with placebo	Combination formulation prevents attribution of effects solely to glutathione	Moderate
Etnawati *et al*. (2019)	Controlled clinical study	Indonesian women (*n* = 74)	Topical	GSH-containing skin-care products	8 weeks	Melanin index and skin tone assessment	Mild but statistically significant depigmenting effect	Cosmetic formulation heterogeneity and short follow-up	Low–Moderate
Richie *et al*. (2015)	Randomized double-blind trial	Healthy adults (*n* = 54)	Oral	GSH 250 mg/day or 1000 mg/day	6 months	Whole blood glutathione concentrations and oxidative stress biomarkers	Increased body glutathione stores and improved redox parameters, supporting oral bioavailability	Not designed to assess skin-lightening outcomes	Moderate
Solnier *et al*. (2026)	Randomized crossover clinical trial	Healthy adults (*n* = 14)	Oral	Oral GSH supplementation	Short-term crossover study	Plasma glutathione metabolites and bioavailability markers	Demonstrated measurable oral bioavailability and favorable safety profile	No pigmentation-related endpoints	Moderate

Abbreviations: GSH, glutathione; GSSG, oxidized GSH; IV, intravenous

The relevant literature should be considered alongside evidence for GSH-based skin-lightening interventions in general clinical practice. While RCTs and observational studies have assessed oral and topical formulations, few studies to date have specifically examined IV GSH. The current enthusiasm for IV administration, therefore, has largely stemmed from theoretical pharmacokinetic benefits and anecdotal clinical experience rather than solid comparative evidence.

IV GSH treatment has little clinical evidence for skin lightening, with only a handful of published studies examining this intervention. Zubair *et al*. [19] conducted the most extensive study to date, an RCT comparing IV GSH plus vitamin C with placebo in Pakistani patients (enrolled 50, completed 32) seeking skin lightening. The treatment regimen was 1200 mg of IV GSH with vitamin C, given twice weekly for 6 weeks. Clinical assessment and subjective satisfaction of outcome measures were the primary outcomes, such as improvement in skin tone.

Results showed that 37.5% of patients in the treatment group observed significant improvement compared to baseline, whereas this was insignificant in the placebo group. Yet, with the dosing regimen, the study also reported some concerning adverse events in liver function, for example, liver function test derangements in eight patients and one case of anaphylaxis, suggesting serious safety issues [19]. Despite being among the few RCTs in this field, the study is constrained by low sample size, short follow-up period, and non-standard outcome measures. Also, the subjective nature of the assessments, combined with a lack of standard objective measures, calls into question measurement bias and the efficacy of the reported benefits in clinic.

Katipunan and Villafuerte [20] conducted an earlier pilot study in the Philippines examining weekly IV administration of 600 mg reduced GSH. This study used objective measurement of skin lightening via melanin index readings obtained with narrow-band reflectance spectrophotometry. Participants received weekly injections for several weeks, followed by melanin index measurements at various body sites (face, arms, and trunk). The results showed statistically significant reductions in melanin index values across most measurement sites, and the treatment was generally well tolerated. However, because they did not include a placebo control group, the researchers could not clearly separate treatment effects from natural fluctuations in results or placebo responses. Moreover, follow-up duration was insufficient to determine the extent of skin-lightening effects following cessation of treatment [20].

Taken together, these early studies indicate that IV GSH may have a depigmenting effect in a small number of individuals. The size and persistence of these effects are unknown, and the available studies are insufficient to distinguish true treatment effects from placebo responses, spontaneous variation in pigmentation, or confounding factors such as sun exposure and concomitant use of other depigmenting agents.

Among existing IV GSH studies, a key limitation is the lack of standardized outcome assessments. Some investigations rely on subjective clinical assessment or patient-reported outcomes, while others use objective colorimetry or melanin index measurements. The Taylor hyperpigmentation scale, Mexameter readings, and spectrophotometric melanin indices have all been used, but there is no consensus on which is best suited to clinically meaningful change [17]. Furthermore, most studies focused on relatively short treatment durations, typically ranging from 4 to 12 weeks, with limited long-term follow-up and evaluation for durability of effects or delayed adverse events. IV GSH dosing regimens have also been extremely variable according to the published studies, ranging from 500 mg to 1200 mg per session and taken either weekly or twice weekly [19,20]. No dose-response studies have been published to establish optimal dosing.

Collectively, the available clinical studies are heterogeneous in design, dosing regimens, and outcome assessment methods, making direct comparison difficult. Most small studies are underpowered, and inadequate control-group data are available to draw definitive conclusions about efficacy. While the evidence is very preliminary, the whole available clinical evidence is not enough to confirm uniform, reproducible skin-lightening efficacy of IV GSH.

Given these limitations, interpretation of individual studies in isolation may be misleading. Systematic reviews and broader evaluations of GSH-based treatments provide valuable context by synthesizing results from different preparations, study designs, and patient populations, but they also highlight common methodological deficiencies (Table 1). A structured review of systemic GSH reported statistically significant reductions in melanin indices across a variety of studies [24], but highlighted that overall evidence was low to moderate and that clinical benefits were generally modest. Similarly, Sarkar *et al*. [11], in a systematic review, concluded that the literature is limited by small samples, diverse study designs and outcomes, and insufficient long-term follow-up, which precludes definitive conclusions about efficacy. These observations have largely been supported by more recent reviews and clinical investigations. Gupta [10] shared the dermatological view of GSH formulations and emphasized that the evidence for IV administration is much weaker than for oral formulations. Additional clinical studies [39–42] generally showed mild-to-moderate improvements in skin pigmentation; however, treatment responses were variable and often limited by small study populations, short treatment durations, a lack of objective outcome measures, and insufficient long-term safety assessment (Table 1).

Importantly, Davids *et al*. [23] highlighted several population-specific considerations, particularly in individuals with darker skin phototypes, including the potential risk of paradoxical post-inflammatory hyperpigmentation and the limited generalizability of findings derived predominantly from Asian study populations. These concerns further underscore the need for caution when extrapolating currently available data to broader and more ethnically diverse populations.

However, a biologically plausible pathway does not necessarily translate into a clinically meaningful benefit, and the available data do not confirm that the improved skin-lightening benefit from higher systemic exposure is equivalent to, or superior to, the skin-lightening outcome achieved by this route.

### Safety profile of GSH-based skin-lightening interventions

Safety data for GSH-based skin-lightening interventions remain limited and considerably less robust than efficacy data. Most available studies were primarily designed to evaluate efficacy and therefore lacked sufficient power or duration to adequately assess adverse events and long-term safety outcomes. Reported adverse effects vary by route of administration.

Oral and topical GSH formulations have generally demonstrated favorable short-term tolerability compared with IV administration, which raises greater safety concerns because of the higher systemic exposure achieved and the occurrence of potentially serious adverse events. Importantly, much of the currently available safety evidence originates from small clinical studies, case reports, observational investigations, and regulatory communications rather than large prospective safety studies. Consequently, the true incidence of adverse events, particularly following long-term or repeated administration, remains uncertain. Table 2 presents a comparative summary of reported adverse effects associated with different GSH formulations.

**Table 2:** Reported and potential adverse effects of GSH used for skin lightening

Adverse Event	Route of Administration	Source of Evidence	Frequency / Severity	Comments	Level of Evidence
Gastrointestinal discomfort (bloating, abdominal discomfort, nausea, loose stools)	Oral	Arjinpathana & Asawanonda (2012); Richie *et al*. (2015); Sitohang & Ninditya (2020)	Mild; uncommon	Usually self-limiting and rarely requires treatment discontinuation	Moderate
Headache and transient dizziness	Oral	Handog *et al*. (2016); Richie *et al*. (2015)	Mild and infrequent	No serious sequelae reported; symptoms resolved spontaneously	Low–Moderate
Local irritation, erythema, dryness, pruritus	Topical	Watanabe *et al*. (2014); Etnawati *et al*. (2019); Khanna *et al*. (2025)	Mild and transient	Topical glutathione preparations were generally well tolerated	Moderate
Infusion-related reactions (nausea, flushing, dizziness, malaise)	IV	Katipunan & Villafuerte (2011); observational reports	Mild to moderate; incidence unknown	Usually transient but poorly documented owing to limited safety reporting in IV studies	Low
Hypersensitivity reactions including anaphylaxis	IV	Zubair *et al*. (2016); regulatory communications	Rare but potentially serious	Potentially life-threatening; true incidence remains uncertain because of limited sample sizes	Low–Moderate
Elevated liver enzymes/hepatotoxicity	IV	Zubair *et al*. (2016); case reports	Rare; causality uncertain	Concomitant administration of vitamin C and other agents may have confounded findings; reversibility incompletely documented	Low
Systemic inflammatory response syndrome (SIRS)	IV	Sharma *et al*. (2025) case report	Extremely rare but severe	Reported following administration of a high-dose glutathione-containing revitalizing solution; causality requires further evaluation	Very Low
Renal dysfunction or altered renal parameters	IV	Regulatory communications and observational reports	Rare	No controlled studies have established a causal relationship	Very Low
Thyroid dysfunction (hypo-/hyperthyroidism)	IV	Regulatory reports and anecdotal observations	Rare; causal relationship uncertain	Long-term endocrine consequences remain poorly characterized	Very Low
Infectious complications related to IV administration (phlebitis, contamination risks)	IV	Regulatory advisories (Philippine FDA, Saudi FDA)	Unknown	Potential risks associated with repeated injections and unregulated cosmetic administration	Very Low
Paradoxical dyschromia or uneven pigmentation	Any formulation, particularly in darker phototypes	Davids *et al*. (2016); Sonthalia *et al*. (2016)	Theoretical/rare	Alterations in melanogenesis may potentially result in uneven pigmentation responses	Very Low
Potential increased photosensitivity and altered melanoprotection due to pheomelanin predominance	Long-term systemic exposure	Villarama & Maibach (2005); Weschawalit *et al*. (2017)	Theoretical only	No direct clinical evidence currently supports increased skin cancer risk or photosensitivity	Very Low
Unknown long-term systemic safety	All formulations	Sitohang & Ninditya (2020); Sarkar *et al*. (2025); Gupta (2025)	Unknown	Most studies had follow-up periods of only 4–12 weeks, preventing assessment of long-term safety outcomes	Very Low
Regulatory advisories regarding cosmetic use of IV glutathione	IV	Philippine FDA (2019); Saudi FDA (2015)	National safety warnings issued	Authorities warned against cosmetic IV glutathione because of insufficient efficacy data and potential safety concerns	Regulatory Evidence
Regulatory advisories regarding cosmetic use	IV	Philippine FDA (2019); Saudi FDA (2015)	National safety warnings issued	Based on insufficient efficacy and safety data	Regulatory Evidence

Safety remains a major concern surrounding the use of GSH for cosmetic skin lightening, particularly when administered intravenously. Among the reported adverse effects, hepatotoxicity has received considerable attention. In the RCT by Zubair *et al*. [19], eight patients receiving IV GSH (1200 mg) in combination with vitamin C twice weekly showed increased liver enzyme levels. While these abnormalities were transient, there was little, if any, documentation about the reversibility and long-term effects. Because GSH is a key endogenous antioxidant and hepatoprotective molecule, the underlying mechanism remains undetermined. Possible causes include dose-related changes in metabolism, individual susceptibility, or coadministration of compounds such as vitamin C [19]. Severe hypersensitivity reactions should also be considered. Zubair *et al*. [19] reported one incidence of anaphylaxis during treatment, emphasizing that even though the occurrence of these reactions is rare, they can be fatal. As a result, IV GSH should be given only in conditions in which prompt medical treatment is available – such as when severe allergic reactions need to be promptly handled.

Case reports, toxicological analyses, and regulatory communications have raised additional concerns. Thyroid dysfunction, including both hypo- and hyperthyroidism, has been described in individuals receiving IV GSH for cosmetic purposes, although a definite causal relationship has not been established [10,16]. Similarly, alterations in renal function have been reported sporadically, but the incidence, mechanisms, and clinical significance of these findings remain poorly defined [11].

By comparison, oral and topical GSH preparations have generally demonstrated a more favorable short-term safety profile. Most reported adverse events have been mild and self-limiting, including transient gastrointestinal discomfort, headache, dizziness, and minor local skin irritation [18,39,40,43]. However, most oral and topical studies have been relatively short, typically ranging from four to twelve weeks, limiting conclusions about long-term safety [11,24].

Additional evidence from pharmacological and experimental studies provides further context. Hauser *et al*. [44], in a pilot study evaluating IV GSH in patients with Parkinson’s disease, reported variable tolerability with high-dose IV administration, suggesting this route may carry safety considerations beyond those of oral supplementation. In contrast, Richie *et al*. [36] reported satisfactory tolerability of oral GSH in healthy subjects over 6 months, which is not easily transferable to IV treatment because these patients have radically different pharmacokinetics and plasma concentration peaks. Furthermore, studies reported that oral GSH is significantly more metabolically degraded and that systemic exposure is much lower than with IV administration [45,46].

Preclinical studies likewise advocate for a conservative approach. Additionally, speculation suggests that long-term changes in melanogenesis may occur. As GSH induces a switch from eumelanin to pheomelanin production, sustained changes in pigment composition may alter vulnerability to oxidative damage and photocarcinogenesis; however, current clinical studies have not demonstrated evidence of increased melanoma risk [14,15].

These safety concerns are especially relevant since skin lightening is mostly a cosmetic indication. Where there is no conclusive, consistent evidence of substantial clinical benefit, even rare severe adverse events can significantly shift the overall risk–benefit balance. This uncertainty is further reflected in the precautionary comments of the Philippine Food and Drug Administration [21] and the Saudi Food and Drug Authority [22], which advise caution with respect to IV GSH use for cosmetic purposes. Overall, evidence suggests that oral and topical GSH products are well tolerated in the short term, whereas IV administration raises greater safety concerns and remains insufficiently studied, particularly regarding long-term effects [10,11,24,46].

## COMPARATIVE PERSPECTIVES OF ORAL, TOPICAL, AND IV GSH: BIOAVAILABILITY, CLINICAL EFFECTS, AND RISK–BENEFIT CONSIDERATIONS

**Figure 3:**
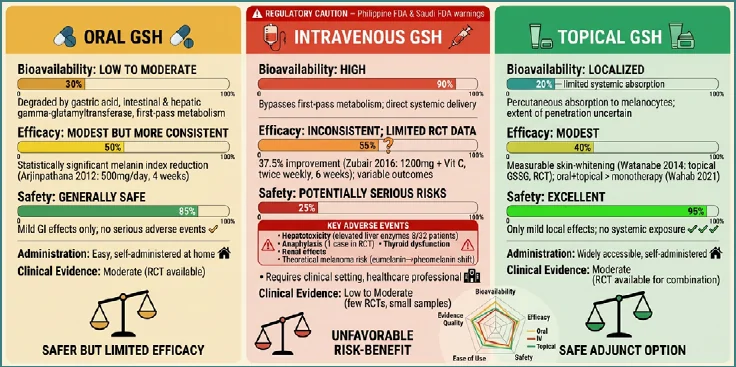
Comparative risk–benefit profiles of oral, topical, and IV GSH formulations for skin lightening. IV GSH achieves the highest systemic bioavailability by bypassing first-pass metabolism but is associated with greater uncertainty regarding long-term safety and potential serious adverse events. Oral and topical formulations demonstrate lower systemic exposure but generally more favorable short-term safety profiles. Current evidence does not conclusively demonstrate superior clinical efficacy of IV administration despite its pharmacokinetic advantages. **Abbreviations: FDA, Food and Drug Administration; GI, gastrointestinal; GSSG, oxidized GSH; IV, intravenous**.

The route of GSH administration substantially influences its pharmacokinetic profile, clinical effects, and safety considerations (Table 3, Figure 3). However, oral supplementation has been investigated more extensively than IV administration in cosmetic skin lightening.

Some clinical trials show slight beneficial effects of oral GSH. In a randomized, double-blind, placebo-controlled trial, Arjinpathana and Asawanonda [43] reported a statistically significant reduction in melanin index after oral GSH, but the magnitude of the effect and its clinical relevance were unclear. Similarly, Handog *et al*. [18] reported reduced facial pigmentation with oral and buccal GSH formulations, without serious adverse events. Additional studies have generally reported mild-to-moderate improvements in skin pigmentation, although treatment responses have been variable and most studies were limited by small sample sizes and short follow-up periods [40-42,46].

**Table 3:** Comparison of oral, topical, and IV GSH for skin lightening

Parameter	Oral GSH	Topical GSH	IV GSH
**Route characteristics**	Systemic administration via gastrointestinal absorption	Local cutaneous administration	Direct systemic administration via intravenous infusion
**Systemic bioavailability**	Traditionally considered low because of gastrointestinal degradation and first-pass metabolism; however, recent studies demonstrate measurable systemic absorption and increased body GSH stores	Minimal systemic absorption; effects are primarily local	Highest systemic exposure owing to bypass of gastrointestinal and hepatic metabolism
**Pharmacokinetic evidence**	Supported by human pharmacokinetic and metabolomic studies demonstrating increased circulating and tissue GSH levels	Limited pharmacokinetic data available	No formal pharmacokinetic studies evaluating skin-lightening outcomes
**Evidence base**	Most extensively studied route, including several randomized controlled trials and bioavailability studies	Limited but growing evidence from controlled clinical studies	Very limited evidence, mainly one pilot study and one small RCT
**Number of clinical studies identified in this review**	Approximately 5–6 studies	Approximately 3 studies	Only 2 studies directly evaluating skin-lightening efficacy
**Clinical efficacy**	Modest but relatively consistent reductions in melanin indices and improvement in skin brightness reported	Mild-to-moderate improvements in skin brightness, pigmentation, and skin condition	Variable and inconsistent efficacy; superiority over oral formulations has not been demonstrated
**Onset of effect**	Generally observed within 4–12 weeks	Usually observed within 8–12 weeks	Uncertain; insufficient evidence to determine whether onset is faster than oral therapy
**Duration of available follow-up**	Up to 6 months in bioavailability studies; most pigmentation studies limited to 4–12 weeks	Predominantly short-term (8–12 weeks)	Predominantly short-term (6–10 weeks)
**Common adverse effects**	Mild gastrointestinal discomfort, headache, bloating, transient dizziness	Mild local irritation, erythema, dryness, pruritus	Infusion-related reactions including nausea, flushing, dizziness, and malaise
**Serious adverse effects reported**	None consistently reported in clinical trials	None consistently reported	Rare hypersensitivity reactions and isolated reports of systemic inflammatory reactions; causality for hepatotoxicity, thyroid, and renal dysfunction remains uncertain
**Long-term safety data**	Limited but generally reassuring	Very limited	Largely unavailable
**Regulatory concerns**	Minimal regulatory concerns	Minimal regulatory concerns	Multiple regulatory advisories issued regarding cosmetic IV use because of insufficient efficacy and safety data
**Quality of evidence**	Moderate for short-term safety and modest efficacy	Low to moderate	Very low to low
**Overall risk-benefit assessment**	Favorable short-term safety profile with modest efficacy	Favorable safety profile with modest efficacy	Uncertain benefit with comparatively greater safety concerns and insufficient long-term evidence
**Current clinical role**	May be considered as an adjunctive cosmetic intervention in selected individuals	Useful adjunctive therapy for selected patients with hyperpigmentation	Should be considered investigational and approached cautiously until robust efficacy and safety data become available

Richie *et al*. [36] further demonstrated that prolonged oral GSH supplementation significantly increased systemic GSH stores without major safety concerns, supporting the biological plausibility of oral administration. However, whether these increases in systemic GSH concentrations translate into clinically meaningful and durable skin-lightening effects remains uncertain.

Topical GSH formulations have also shown encouraging results. Watanabe *et al*. [47] demonstrated modest improvements in skin brightness and pigmentation parameters with excellent short-term tolerability. Because topical preparations primarily exert localized effects and avoid systemic exposure, they may offer a favorable safety profile, although their depigmenting efficacy appears relatively modest. Wahab and colleagues [47] examined combination therapy with oral and topical GSH in a double-blind RCT. Oral and topical GSH showed a greater skin-lightening effect than monotherapy or placebo, as measured by melanin index and colorimetry. This observation suggests that multimodal approaches targeting melanogenesis through diverse pathways are effective, but this study did not include an IV GSH arm for comparison [48].

IV GSH theoretically offers the greatest systemic bioavailability because it bypasses gastrointestinal degradation and first-pass metabolism. Pharmacokinetic considerations therefore suggest that IV administration should achieve substantially higher plasma GSH concentrations than oral or topical formulations [18]. However, an important question remains unanswered: whether these higher circulating concentrations translate into superior clinical outcomes.

Currently, little evidence suggests that IV GSH provides clinically significant efficacy over oral or topical forms. The only randomized study evaluating IV GSH for skin lightening was by Zubair *et al*. [19]; they found that pigmentation improved somewhat but also reported potentially serious adverse events, including elevated liver enzymes and anaphylaxis. In addition, in the current study, concurrent vitamin C use makes it challenging to ascertain the independent effect of GSH on the observed effects.

The relative risk–benefit profiles of various GSH formulations vary, therefore, greatly (Figure 3). Oral and topical versions generally have good short-term safety profiles; adverse events are usually mild gastrointestinal symptoms or local skin irritation [36,39,43,47,48,49]. IV administration, however, is more dangerous due to serious hypersensitivity reactions, hepatotoxicity, endocrine disturbances, and other long-term poorly characterized effects [10,19,23].

Clinically, formulation selection balances theoretical pharmacological utility (pharmacological benefits) with efficacy and safety, and it is the basis for deciding which formulations to use. While IV administration achieves the highest plasma concentrations, evidence remains insufficient to conclude that this route yields superior or more durable skin-lightening effects. On the other hand, oral and topical preparations seem to yield a more favorable short-term safety profile, but with generally modest efficacy. As a result, routine cosmetic use of IV GSH in clinical practice should remain limited to highly supervised clinical centers or appropriate research studies. Additional large-scale randomized trials that directly compare various routes of administration are needed before specific evidence-based recommendations can be determined.

### Vitamin C as an adjunct and a major confounding factor in clinical studies

Before discussing the limitations of the current evidence, it is important to consider the role of vitamin C, which is frequently co-administered with IV GSH and represents a significant source of methodological confounding in the available literature.

In clinical practice, vitamin C is frequently administered with IV GSH because of their proposed complementary antioxidant and depigmenting effects. Several biochemical mechanisms support this combination. Vitamin C inhibits melanogenesis by reducing dopaquinone back to L-DOPA, thereby interfering with a key step in melanin synthesis [25,50,51]. In addition, ascorbic acid helps maintain cellular redox homeostasis and may facilitate regeneration of GSH from GSSG, potentially sustaining intracellular concentrations of biologically active GSH [13,52,53].

The confounding effect of vitamin C co-administration represents one of the major methodological limitations in the existing literature and has not been adequately addressed in most studies. The RCT by Zubair *et al*. [19], which remains one of the most frequently cited clinical studies evaluating IV GSH for skin lightening, administered IV GSH exclusively in combination with vitamin C. Consequently, it is impossible to determine whether the reported clinical improvement or the observed adverse events were attributable to GSH, vitamin C, or their combined effects.

This issue is particularly important because vitamin C itself possesses well-established anti-melanogenic properties. In addition to its antioxidant effects, vitamin C can directly modulate tyrosinase activity and reduce oxidative stress-mediated stimulation of melanogenesis [50,54]. Therefore, the approximately 37.5% improvement reported by Zubair *et al*. [19] cannot be confidently attributed to IV GSH alone.

From a pharmacokinetic perspective, IV vitamin C achieves plasma concentrations substantially higher than those obtained through oral supplementation because intestinal absorption becomes saturated at relatively low oral doses [51,55,56]. High-dose IV vitamin C has generally demonstrated an acceptable safety profile in several clinical settings, including oncology and critical illness, although potential adverse effects such as oxalate nephropathy and hemolysis in individuals with glucose-6-phosphate dehydrogenase deficiency have been reported [55–58].

Despite its widespread incorporation into cosmetic infusion protocols, no high-quality evidence currently demonstrates that vitamin C provides additive or synergistic clinical benefits when combined with IV GSH. Likewise, no adequately powered studies have directly compared IV GSH alone, vitamin C alone, and combination therapy.

This important evidence gap has also been emphasized in recent systematic reviews. Sitohang and Ninditya [24] also reported heterogeneity across GSH studies and argued that interpretation of combination regimen results was complex. Similarly, Sarkar *et al*. [11] found that the existing literature remains inadequate to assess the independent efficacy of IV GSH because of methodological constraints, small sample sizes, and the common use of concomitant therapies.

Altogether, the evidence to date indicates that the justification for combining vitamin C with IV GSH remains largely theoretical and mechanistically driven rather than empirically established. Subsequent clinical trials might thus consider independent treatment arms for GSH monotherapy, vitamin C monotherapy, or combination therapy, together with standardized objective pigmentation assessments and longer follow-up periods. Such studies are necessary to assess whether the potential biochemical synergy of these agents leads to clinically meaningful improvements in skin pigmentation.

## LIMITATIONS OF THE CURRENT EVIDENCE AND FUTURE RESEARCH DIRECTIONS

Although interest in the potential use of GSH for skin lightening has increased, the recent evidence base is sparse and should be treated with caution. In general, the evidence base supporting IV GSH is limited to low to moderate levels because of small sample sizes, methodological heterogeneity, and inconsistent findings [11,24].

Most published studies included fewer than 50 participants and were conducted predominantly in Asian populations, limiting the generalizability of the findings to other ethnic groups and skin phototypes [23]. Significant variations also exist in dosing regimens, treatment duration, frequency of administration, and methods for evaluating pigmentation outcomes, making direct comparisons between studies problematic. The follow-up time, which was generally short (during treatment or shortly after), is another critical limitation. Thus, it is unclear how long clinical effects last and whether repeated GSH injections are safe in the long term. Frequent co-administration of vitamin C also constitutes a significant confounding factor, making it challenging to evaluate the independent efficacy of GSH.

No direct comparative studies have compared IV, oral, and topical formulations. Accordingly, future studies should aim for adequately powered RCTs that include standardized outcomes, prolonged follow-up periods, separate monotherapy and combination treatment arms, and close surveillance of hepatic, renal, and endocrine safety.

Overall, a substantial gap remains between the widespread clinical use of IV GSH and the quality of evidence supporting its efficacy and safety.

## CONCLUSION

GSH has become popular as a cosmetic skin-lightening agent, especially in IV preparations; however, high-quality clinical evidence remains scarce. While many biochemical pathways might underlie its depigmenting effects, such as modulating tyrosinase activity, antioxidant activity, and stimulation of pheomelanin production, none of these mechanisms has reliably led to clinically meaningful responses. Current evidence is limited by small sample sizes, heterogeneous study designs, short follow-up durations, and variable outcome measures, making conclusions about effectiveness difficult. Frequent co-administration of vitamin C is also a major confounding factor that complicates interpretation of the available data. Safety concerns regarding IV administration, including hepatotoxicity, hypersensitivity reactions, and possible renal and endocrine effects, necessitate cautious use given the primarily cosmetic indication for treatment. As it stands, IV GSH without a strong recommendation for skin lightening is not a recommended regular practice today. Clinicians must carefully counsel patients about the uncertain benefits and potential risks. Oral and topical formulations seem to have better safety profiles, though their depigmenting effects are still modest. Evidence-based recommendations for IV GSH are required only based on well-designed, adequately powered RCTs with long-term safety assessment.

## References

[1] Bento-Lopes L, Cabaço LC, Charneca J, Neto MV, Seabra MC, Barral DC (2023). Melanin’s journey from melanocytes to keratinocytes: uncovering the molecular mechanisms of melanin transfer and processing. Int J Mol Sci.

[2] Videira IF, Moura DF, Magina S (2013). Mechanisms regulating melanogenesis. An Bras Dermatol.

[3] Schlessinger DI, Rahimi N, Schlessinger J (2026). Biochemistry, melanin. StatPearls [Internet].

[4] Dadzie OE, Petit A (2009). Skin bleaching: highlighting the misuse of cutaneous depigmenting agents. J Eur Acad Dermatol Venereol.

[5] Davids LM, van Wyk J, Khumalo NP, Jablonski NG (2016). The phenomenon of skin lightening: is it right to be light?. S Afr J Sci.

[6] Pollock S, Taylor S, Oyerinde O, Nurmohamed S, Dlova N, Sarkar R (2021). The dark side of skin lightening: an international collaboration and review of a public health issue affecting dermatology. Int J Womens Dermatol.

[7] Gan C, Rodrigues M (2024). An update on new and existing treatments for the management of melasma. Am J Clin Dermatol.

[8] Carneiro R, Lopes CM, Amaral MH (2026). Innovative skin depigmenting strategies: a review. Appl Sci.

[9] Alzahrani TF, Alotaibi SM, Alzahrani AA, Alzahrani AF, Alturki LE, Alshammari MM (2025). Exploring the safety and efficacy of glutathione supplementation for skin lightening: a narrative review. Cureus.

[10] Gupta S (2025). Glutathione in dermatology: a bright future or fading hype?. Cosmoderma.

[11] Sarkar R, Yadav V, Yadav T, Janaani P, Mandal I (2025). Glutathione as a skin-lightening agent and in melasma: a systematic review. Int J Dermatol.

[12] Pizzorno J (2014). Glutathione! Integr Med (Encinitas).

[13] Georgiou-Siafis SK, Tsiftsoglou AS (2023). The key role of GSH in keeping the redox balance in mammalian cells: mechanisms and significance of GSH in detoxification via formation of conjugates. Antioxidants (Basel).

[14] Villarama CD, Maibach HI (2005). Glutathione as a depigmenting agent: an overview. Int J Cosmet Sci.

[15] Chang TS (2009). An updated review of tyrosinase inhibitors. Int J Mol Sci.

[16] Sonthalia S, Daulatabad D, Sarkar R (2016). Glutathione as a skin whitening agent: facts, myths, evidence and controversies. Indian J Dermatol Venereol Leprol.

[17] Sonthalia S, Jha AK, Lallas A, Jain G, Jakhar D (2018). Glutathione for skin lightening: a regnant myth or evidence-based verity?. Dermatol Pract Concept.

[18] Handog EB, Datuin MSL, Singzon IA (2016). An open-label, single-arm trial of the safety and efficacy of a novel preparation of glutathione as a skin-lightening agent in Filipino women. Int J Dermatol.

[19] Zubair S, Hafeez S, Mujtaba G (2016). Efficacy of intravenous glutathione vs. placebo for skin tone lightening. J Pak Assoc Dermatol.

[20] Katipunan KK, Villafuerte LL (2011). Intravenous glutathione as a skin lightening agent: a pilot study on its efficacy and safety. J Philipp Dermatol Soc.

[21] Food and Drug Administration Philippines FDA Advisory No 2019182: Unsafe use of glutathione as skin lightening agent. https://www.fda.gov.ph/fda-advisory-no-2019-182-unsafe-use-of-glutathione-as-skin-lightening-agent/.

[22] Saudi Food and Drug Authority (2015). SFDA warns from skin-whitening injections. https://www.sfda.gov.sa/en/warnings/1347.

[23] Davids LM, van Wyk JC, Khumalo NP (2016). Intravenous glutathione for skin lightening: inadequate safety data. S Afr Med J.

[24] Sitohang IBS, Ninditya S (2020). Systemic glutathione as a skin-whitening agent in adult. Dermatol Res Pract.

[25] Li C, Kuai L, Cui R, Miao X (2022). Melanogenesis and the targeted therapy of melanoma. Biomolecules.

[26] Jara JR, Aroca P, Solano F, Martinez JH, Lozano JA (1988). The role of sulfhydryl compounds in mammalian melanogenesis: the effect of cysteine and glutathione upon tyrosinase and the intermediates of the pathway. Biochim Biophys Acta.

[27] Snyman M, Walsdorf RE, Wix SN, Gill JG (2024). The metabolism of melanin synthesis—from melanocytes to melanoma. Pigment Cell Melanoma Res.

[28] Bickers DR, Athar M (2006). Oxidative stress in the pathogenesis of skin disease. J Invest Dermatol.

[29] Hong S, Lim H, Kim DH (2026). Biological roles of melanin and natural product-derived approaches for its modulation. Int J Mol Sci.

[30] Benathan M (1997). Opposite regulation of tyrosinase and glutathione peroxidase by intracellular thiols in human melanoma cells. Arch Dermatol Res.

[31] Benathan M, Virador V, Furumura M, Kobayashi N, Panizzon RG, Hearing VJ (1999). Co-regulation of melanin precursors and tyrosinase in human pigment cells: roles of cysteine and glutathione. Cell Mol Biol (Noisy-le-grand).

[32] Nagapan TS, Lim WN, Basri DF, Ghazali AR (2019). Oral supplementation of L-glutathione prevents ultraviolet B-induced melanogenesis and oxidative stress in BALB/c mice. Exp Anim.

[33] Witschi A, Reddy S, Stofer B, Lauterburg BH (1992). The systemic availability of oral glutathione. Eur J Clin Pharmacol.

[34] Wei T, Thakur SS, Liu M, Wen J (2022). Oral delivery of glutathione: antioxidant function, barriers and strategies. Acta Materia Medica.

[35] Solnier J, Du M, Zhang Y, Roh YS, Kuo YC, Ibi A (2026). A targeted metabolomic assessment of oral glutathione bioavailability and safety in humans: a randomized crossover clinical trial. Antioxidants (Basel).

[36] Richie JP, Nichenametla S, Neidig W, Calcagnotto A, Haley JS, Schell TD (2015). Randomized controlled trial of oral glutathione supplementation on body stores of glutathione. Eur J Nutr.

[37] Wang F, Ma W, Fan D, Hu J, An X, Wang Z (2024). The biochemistry of melanogenesis: an insight into the function and mechanism of melanogenesis-related proteins. Front Mol Biosci.

[38] Fu C, Chen J, Lu J, Yi L, Tong X, Kang L (2020). Roles of inflammation factors in melanogenesis (Review). Mol Med Rep.

[39] Duperray J, Sergheraert R, Chalothorn K, Tachalerdmanee P, Perin F (2022). The effects of the oral supplementation of L-cystine associated with reduced L-glutathione-GSH on human skin pigmentation: a randomized, double-blinded, benchmark-and placebo-controlled clinical trial. J Cosmet Dermatol.

[40] Etnawati K, Adiwinarni DR, Susetiati DA, Sauchi Y, Ito H (2019). The efficacy of skin care products containing glutathione in delivering skin lightening in Indonesian women. Dermatol Reports.

[41] Mohan S, Mohan L, Sangal R, Singh N (2020). Glutathione for skin lightening for dermatologists and cosmetologists. Int J Res Dermatol.

[42] Sharma D, Daram S, Sharma P (2025). Systemic inflammatory response syndrome following high-dose intravenous glutathione-containing revitalising solution in a patient on tirzepatide: a case report. Cureus.

[43] Arjinpathana N, Asawanonda P (2012). Glutathione as an oral whitening agent: a randomized, double-blind, placebo-controlled study. J Dermatolog Treat.

[44] Hauser RA, Lyons KE, McClain T, Carter S, Perlmutter D (2009). Randomized, double-blind, pilot evaluation of intravenous glutathione in Parkinson’s disease. Mov Disord.

[45] Chirumbolo S, Franzini M, Ricevuti G, Valdenassi L (2025). Intravenous glutathione should not be mismatched with ozone as an antioxidant therapy. Biomed Pharmacother.

[46] Naik SB, Mahabala C, Alva V, Jyothi CS (2025). Is intravenous glutathione therapy as skin whitening agent safe? A clinical experience. Muller J Med Sci Res.

[47] Watanabe F, Hashizume E, Chan GP, Kamimura A (2014). Skin-whitening and skin-condition-improving effects of topical oxidized glutathione: a double-blind and placebo-controlled clinical trial in healthy women. Clin Cosmet Investig Dermatol.

[48] Wahab S, Anwar AI, Zainuddin AN, Hutabarat EN, Anwar AA, Kurniadi I (2021). Combination of topical and oral glutathione as a skin-whitening agent: a double-blind randomized controlled clinical trial. Int J Dermatol.

[49] Khanna R, Rambhia P, Chapas A (2025). Systematic review of the efficacy and safety of topical glutathione in dermatology. J Clin Aesthet Dermatol.

[50] Panich U, Tangsupa-a-nan V, Onkoksoong T, Kongtaphan K, Kasetsinsombat K, Akarasereenont P (2011). Inhibition of UVA-mediated melanogenesis by ascorbic acid through modulation of antioxidant defense and nitric oxide system. Arch Pharm Res.

[51] Weschawalit S, Thongthip S, Phutrakool P, Asawanonda P (2017). Glutathione and its antiaging and antimelanogenic effects. Clin Cosmet Investig Dermatol.

[52] Smirnoff N (2001). L-ascorbic acid biosynthesis. Vitam Horm.

[53] Lee E, Park HY, Kim SW, Kim J, Lim K (2023). Vitamin C and glutathione supplementation: a review of their additive effects on exercise performance. Phys Act Nutr.

[54] Sanadi RM, Deshmukh RS (2020). The effect of vitamin C on melanin pigmentation — a systematic review. J Oral Maxillofac Pathol.

[55] Padayatty SJ, Sun H, Wang Y, Riordan HD, Hewitt SM, Katz A (2004). Vitamin C pharmacokinetics: implications for oral and intravenous use. Ann Intern Med.

[56] Alangari A, Arif J, Al Qureshah F, Alkhodairy F (2026). Clinical benefits and risks of high-dose intravenous vitamin C: a systematic review. J Med Life.

[57] Chen Q, Espey MG, Sun AY, Pooput C, Kirk KL, Krishna MC (2008). Pharmacologic doses of ascorbate act as a prooxidant and decrease growth of aggressive tumor xenografts in mice. Proc Natl Acad Sci U S A.

[58] Yanase F, Fujii T, Naorungroj T, Belletti A, Luethi N, Carr AC (2020). Harm of IV high-dose vitamin C therapy in adult patients: a scoping review. Crit Care Med.

